# Interprofessional learning in the topical workshops at the Universität zu Lübeck – a qualitative study

**DOI:** 10.3205/zma001788

**Published:** 2025-11-17

**Authors:** Marie Jacob, Kerstin Lüdtke, Katharina Röse

**Affiliations:** 1 Universität zu Lübeck, Institute of Health Sciences, Department of Occupational Therapy, Lübeck, Germany; 2 Universität zu Lübeck, Institute of Health Sciences, Department of Physiotherapy, Lübeck, Germany

**Keywords:** interprofessional relations, learning experience, vocational education, qualitative research, focus group interviews, competency-based education

## Abstract

**Background:**

Interprofessional healthcare requires interprofessional learning (IPL) starting during academic education. The aim of this study is to examine the implementation of IPL in the modules “Topical Workshop (TW) for Orthopaedic Rehabilitation” and “TW for Paediatrics and Child and Adolescent Psychosomatics” at the Universität zu Lübeck (UzL) from the perspective of students in occupational therapy, speech therapy, and physiotherapy.

**Methods:**

This qualitative study was conducted in two phases. In phase 1, qualitative data was collected and analysed using Kuckartz’s method of content-structuring qualitative content analysis. Building on this, two focus group interviews were conducted in phase 2 and analysed using the same method. The data was integrated using a triangulation approach. A final member reflection was carried out.

**Results:**

The analysis of five interviews, five written pre-understandings, the central evaluation, and two focus group interviews shows: Joint learning of new content, understanding of profession-specific content, and the acquisition of interprofessional skills are at the core of IPL. In the modules, the various professions were involved to different extents, approaches to interprofessional collaboration (IPC) were demonstrated, and practical relevance was established. Facilitating factors such as practical content and group work were contrasted by challenges such as a lack of exchange and unsuitable tasks. The perceived learning gains resulted in wishes and ideas for shaping future TWs.

**Conclusion:**

Both facilitating and inhibiting factors for IPL were identified. These lead to approaches for further developing the modules, which can be applied to similar academic contexts.

## Zusammenfassung

**Hintergrund:**

Eine interprofessionelle Gesundheitsversorgung bedarf des interprofessionellen Lernens (ipL) bereits im Studium. Ziel der Studie ist es, die Umsetzung des ipL in den Modulen „Profilwerkstatt (PW) für orthopädische Rehabilitation“ sowie „PW für Pädiatrie und Kinder- und Jugend-Psychosomatik“ an der Universität zu Lübeck aus Sicht von Studierenden der Ergotherapie, Logopädie und Physiotherapie zu untersuchen.

**Methodik:**

Die qualitative Studie erfolgte in zwei Phasen. In Phase 1 wurden qualitative Daten erhoben und mittels inhaltlich strukturierender qualitativer Inhaltsanalyse nach Kuckartz ausgewertet. Aufbauend wurden in Phase 2 zwei Fokusgruppeninterviews geführt und nach demselben Verfahren analysiert. Die Daten wurden mittels der Vorgehensweise der Triangulation miteinander verknüpft. Abschließend erfolgte eine Member Reflection.

**Ergebnisse:**

Die Analyse von fünf Interviews, fünf schriftlich festgehaltenen Vorannahmen, der zentralen Evaluation und zwei Fokusgruppeninterviews zeigt: Gemeinsames Erlernen neuer Inhalte, Verständnis für professionsspezifische Inhalte und der Erwerb interprofessioneller Fähigkeiten stehen im Zentrum des ipL. In den Modulen wurden Professionen unterschiedlich stark einbezogen, Ansätze zur Zusammenarbeit der Professionen aufgezeigt und Praxisbezüge geschaffen. Förderliche Faktoren wie praktische Inhalte und Gruppenarbeiten standen Herausforderungen wie fehlendem Austausch und ungeeignete Aufgaben gegenüber. Der wahrgenommene Lernzuwachs resultiert in Wünschen und Ideen für die Gestaltung zukünftiger PW.

**Schlussfolgerung:**

Es wurden fördernde und hemmende Bedingungen für ipL identifiziert. Daraus ergeben sich Ansätze zur Weiterentwicklung der Module, die auf vergleichbare hochschulische Kontexte übertragbar sind.

## Introduction and objectives

The complex treatment of patients affected by multimorbidity requires coordinated care processes in which various professional groups not only work in parallel but actively interlink their perspectives – in the sense of interprofessional rather than merely multiprofessional collaboration [1], [2]. Such processes are supported by interprofessional education, understood as learning with, from, and about each other, as defined by the Centre for the Advancement of Interprofessional Education [3], in order to effectively integrate the competencies of different professions [2]. Studies show that interprofessional education positively influences students’ attitudes and it has the potential to improve treatment quality, even though its direct impact on patient care has not been clearly demonstrated [4], [5], [6], [7], [8]. Findings from various mixed-methods and qualitative studies indicate that interprofessional teaching enables students to develop a better understanding of other professions’ competencies and strengthens core interprofessional skills such as communication and teamwork [9], [10], [11], [12], [13], [14]. Experiencing diverse roles and recognizing the complementary potential between professions contribute to fostering mutual respect and reinforcing one’s own professional identity [9], [10], [11], [12]. Key success factors include practice-oriented content, the use of simulations, and sufficient time for exchange processes. In contrast, challenges include unequal distribution of professions and differing levels of prior knowledge [11] [12], [13], [15], [16].

Given the growing importance of cross-disciplinary collaboration in healthcare, understanding and further developing IPL within the university context is crucial in preparing future professionals for the complex demands of clinical practice. At the UzL, the shared campus for health-related study programs provides ideal conditions for IPL, which is firmly embedded in the curricula. A central component of IPL at UzL are the TWs – field-specific, interprofessional designed elective modules for students of physiotherapy, occupational therapy, and speech therapy. Students choose from various thematic profiles, including “orthopaedic rehabilitation” and “paediatrics and child and adolescent psychosomatics”. Within these TWs, students deepen their therapeutic competencies through concrete case examples and reflect on their role within the interprofessional team. The modules include a joint lecture series, profile-specific seminars, and a clinical observation placement. The interprofessional orientation of the teaching teams and the curricular integration of the TWs provide a suitable environment for developing interprofessional competencies. Although central evaluations have been conducted by the Office for Quality and Organizational Development at UzL, there is a lack of in-depth insight into students’ subjective experiences with IPL in the TWs. This study aims to address this gap by exploring students’ perspectives and deriving strategies for optimizing the modules. The following research question guides the study: How does IPL take place in the TWs at the UzL from the perspective of physiotherapy, occupational therapy, and speech therapy students? To answer this, the study explores the following sub-questions: What is the students’ subjective understanding of IPL? How is IPL implemented and designed with practical relevance in the TWs and teaching sessions? What learning gains do students perceive in working with other professional groups? What suggestions do students have for improving the TWs?

## Methods

### Study design and research approach

This qualitative, descriptive study explored students’ subjective perspectives on IPL within the TWs [17]. The study followed a constructivist paradigm and employed a range of methods and data sources within a two-phase, iterative research process conducted over several academic semesters [18]. A student research project was integrated into the study as part of the teaching framework. Triangulation was achieved by integrating multiple methods, data sources, and perspectives to ensure comprehensive and credible results [18]. The methodological approach aligns with the core criteria of qualitative research and adheres to the Standards for Reporting Qualitative Research (SRQR) [19], [20]. The study complied with the requirements of the European General Data Protection Regulation (GDPR). Ethical approval was obtained from the Ethics Committee of the UzL (reference number 2022-407). Informed consent and participant confidentiality were ensured throughout the study.

### Data collection

**Figure 1:**
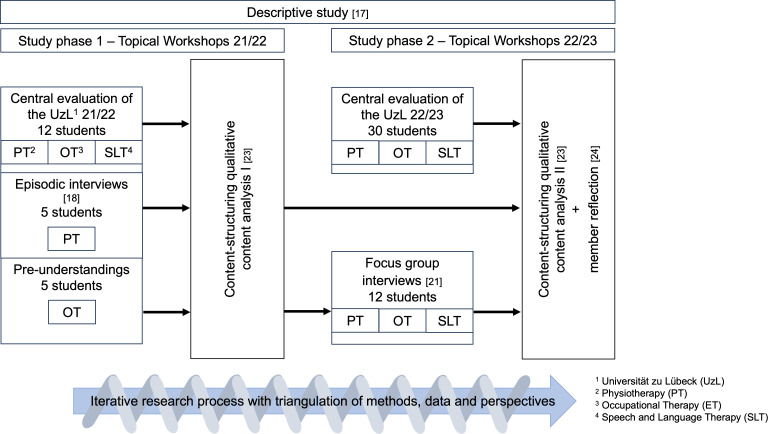
Stages of the research process

In phase 1, as part of a student research project, episodic interviews were conducted by occupational therapy and speech therapy students with five physiotherapy students. Since the study focused exclusively on the TWs from the 2021/2022 academic year, only physiotherapy students were available for interviews. The occupational therapy and speech therapy students had participated in the TWs themselves and were therefore excluded due to their role as researchers. Additionally, the students of occupational therapy and speech therapy reflected on and documented their own pre-understandings to ensure research-practical reflexivity and transparency. These were regularly revisited throughout the research process. As these pre-understandings also represented subjective experiences of IPL in the TWs, five written student pre-understandings were included as an additional data source in the analysis. In phase 2, the first author conducted two focus group interviews involving students from all three therapy professions. A total of 45 students were contacted, of whom 12 agreed to participate. The focus group method was chosen to encourage participants to recall shared experiences and to generate insights through group dynamics that go beyond individual perspectives [21]. The discussions were structured using a developed focus group interview guide based on key themes (see attachment 1 ), with open-ended questions designed to elicit narrative-episodic accounts and conceptual-semantic knowledge [18]. The findings from phase 1 were used to inform the development of the phase 2 focus group interview guide. Each focus group interview lasted approximately 90 minutes and was conducted online via the video conferencing software Webex Teams by Cisco Systems (licensed by UzL). Starting with an introduction round and thematic overview, the discussions evolved based on the perspectives of the three participating professional groups. Before data collection, students completed supplementary questionnaires (see attachment 2 ). In addition, free-text responses from the central UzL evaluation were included: 12 student responses in phase 1 and 30 in phase 2 (see figure 1 (Fig. 1)). The interviews and focus group interviews were fully transcribed verbatim using a content-semantic method, without linguistic smoothing [22].

### Data analysis

The transcripts of the interviews and focus group interviews, the written pre-understandings, and the free-text responses from the central evaluation were treated as independent data sources and analysed individually, using content-structuring qualitative content analysis [23]. Following an iterative process, data from phase 1 were analysed first. After a subsequent round of data collection, phase 2 data were evaluated. Initial coding involved a deductive development of main categories derived from the sub-questions of the research question, followed by the inductive formation of subcategories. The data analysis was conducted successively by two researchers independently to ensure consistency. The finalized coding system was applied to all data from both phases (see attachment 3 ). Finally, a member reflection was conducted with two students from the focus group interviews to discuss the results in greater depth and to gain additional insights [24].

### Researcher characteristics and reflexivity

At the time the study was conducted, the first author was a student who was preparing to take on a future teaching role within the TWs. This dual role brought with it an intrinsic motivation to evaluate and improve the teaching within these modules. Her close proximity to the participants – due to a similar professional training and academic background – potentially enhanced mutual understanding. Ongoing reflection on personal pre-understandings and positionality was carried out to ensure intersubjective transparency in both data collection and analysis. This reflexivity was supported through maintaining a research journal, along with regular discussions with co-authors and other researchers in a research workshop setting. The co-authors are responsible for the modules and serve as instructors in the TWs. They also have extensive experience in research.

## Results

### Sample description

The sample consists of 17 students, including five physiotherapy students (for the episodic interviews) and 12 students for the two focus group interviews (see table 1 (Tab. 1)). The focus group for the Paediatrics TW included three physiotherapy, two occupational therapy, and three speech therapy students, while the focus group for the Orthopaedics TW included two physiotherapy and two occupational therapy students. All participants are state-certified therapists. Additionally, pre-understandings from occupational therapy and speech therapy students, as well as anonymized data from the central evaluations of the UzL, were analysed – though no detailed demographic information was available for these sources.

### Main category 1: Subjective understanding of interprofessional learning

**Figure 2:**
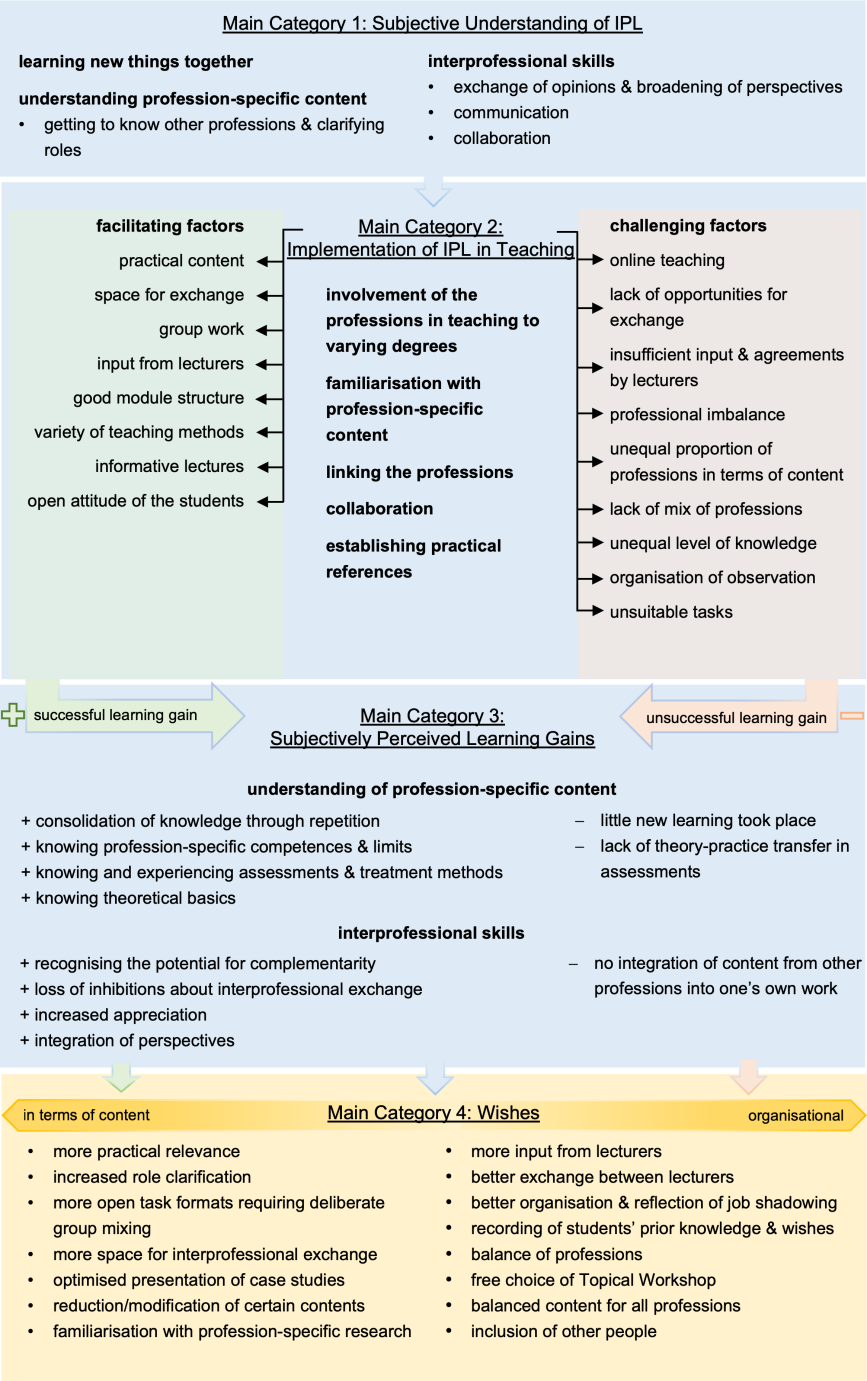
Results overview

The students perceive IPL as an opportunity to acquire new knowledge collaboratively – knowledge that is relevant to all participating professions. One student explained, for example: *“You learn a basic concept, but everyone has a different background, which enriches the learning process”* (F4, lines 256-268). A key emphasis was placed on understanding the specific tasks and techniques of other professions: *“It’s about recognizing the boundaries of your own profession and seeing where the next one begins”* (F1, lines 124-128). In addition to exchanging profession-specific content, students also highlighted the development of interprofessional skills, such as the sharing of experiences and perspectives. One student remarked: *“The exchange of viewpoints helps identify aspects that might have been overlooked within one's own profession and broadens one’s perspective”* (F4, lines 257-268). Moreover, students emphasized the importance of clear communication between professions in order to prevent misunderstandings. The relevance of collaboration was also underscored, characterized by the identification of shared goals and the cooperative handling of challenges. A common objective, according to the students, is to ensure the best possible care for patients – achieved through coordinated collaboration among the various professions: *“We learned that it’s important to set shared goals and work together as a team to achieve them”* (F4, lines 373-378) (see figure 2 (Fig. 2)).

### Main category 2: Implementation of interprofessional learning in teaching

In the TW classes, students reported varying degrees of representation of different professions. For example, in the TW paediatrics course, occupational therapy was perceived as dominant, as reflected in the comment: *“I found it relatively OT-heavy”* (F13, l. 356). In contrast, the TW orthopaedics course was reported to focus more on physiotherapy, with occupational therapy playing a supplementary role. The instructors made efforts to balance these differences by explicitly seeking input from the various professions. One participant noted: *“The instructors always tried to get input from the occupational therapists to highlight both perspectives”* (F2, ll. 208-211). Students reported gaining deeper insights into the specific areas of expertise of the different professions through the TWs. Fundamental theoretical concepts were covered – such as anatomy in orthopaedics and developmental milestones in paediatrics – as well as practical skills like diagnostic testing and specific therapeutic techniques. The practice-oriented nature of the teaching was supported through case studies, treatment simulations, and observational placements. For instance, in the TW paediatrics course, practical scenarios were created through the use of assessments and the simulation of case conferences. In the TW orthopaedics course, students were introduced to the use of assistive devices, which provided hands-on experience with therapeutic interventions.

The students found that practice-oriented teaching methods such as hands-on exercises, job shadowing, and the use of video material were particularly supportive of IPL. A clear structure of the courses and good guidance from the instructors also contributed to successful learning. The interprofessional exchange was perceived as enriching, especially when sufficient time was allocated for discussions and group work. The diversity of teaching methods – ranging from lectures and seminars to practical exercises – also supported learning: *“The mix of different teaching methods, including hands-on exercises and theoretical lectures, was very beneficial”* (E4, ll. 14, 38-40). Challenges were noted in the uneven participation of the different professions and in varying levels of prior knowledge among students. For example, in the TW orthopaedics course, physiotherapy content was reported to dominate, while in the paediatrics course, occupational therapy content was more prevalent. Additionally, a lack of interprofessional exchange was attributed to organizational barriers, such as differing schedules and a lack of shared time for group work (see figure 2 (Fig. 2)).

### Main category 3: Subjectively perceived learning gains

Students reported that the repetition of familiar content helped consolidate their knowledge, which gave them confidence when entering the workforce: *“The opportunity to ask questions and practice helped solidify the knowledge”* (F7, ll. 277-283). They stated that they had gained a better understanding of the specific competencies and limitations of the various professional groups, which, for example, made it easier to refer patients to other specialists. In addition, getting to know and experience the assessments and treatment methods of other professions provided them with practical, hands-on experiences. However, these were not always sufficiently practiced. Some students also mentioned that the high degree of content repetition led to few new insights.

IPL helped students recognize the potential for collaboration and complementarity between the professions: *“It became clear how the professions can complement each other”* (F2, ll. 795-797). Many students reported a reduction in hesitation to engage in interprofessional exchange and felt more confident working with other professions: *“Inhibitions to engage with other professions were reduced”* (F1, ll. 775-778). The increased appreciation for the contributions of other professional groups was supported by a better understanding of their roles and perspectives: *“You gain an even better understanding of the importance of the other professions”* (F2, ll. 795-797). While some students described integrating the perspectives of other professions into their own professional thinking and practice, others noted that they did not incorporate specific content from those professions into their own work (see figure 2 (Fig. 2)).

### Main category 4: Wishes

The students expressed various wishes for improving the IPL. Among these was the desire for more scheduled time for interprofessional exchange within the teaching plan, to promote more intensive collaboration.* “If there had been planned self-study time from the beginning, we probably would have been more motivated to complete the tasks together”* (F10, l. 673-674). A stronger practical orientation was also requested, with concrete suggestions such as more intensive practice of the content:* “It would have been helpful to have more practical exercises in order to really internalize the techniques we learned”* (F7, l. 797-801). Task assignments should be designed more freely, and interprofessional group composition should be ensured to maximize the exchange between different professions. For better understanding and applicability, students wished for optimized case presentations that include realistic details and provide equal information for all professions:* “We need tasks that include all professions so that we can truly learn from each other” *(F13, l. 203-208). Additionally, students wished for more input from instructors, who should share their own experiences with IPC. Better organization and reflection of the observation placements (hospitations) were considered necessary to ensure that all students have the opportunity to gain practical experience in various professions and reflect on it afterward. Gathering students’ prior knowledge and individual preferences before the start of the practical weeks was suggested, to better tailor the teaching to their needs. A particularly unanimous wish was for a balance of representation and content across all professions in the teaching sessions, to enable comprehensive IPL (see figure 2 (Fig. 2)).

## Discussion

This qualitative study explored the subjective perspectives of students in physiotherapy, occupational therapy, and speech and language therapy regarding IPL in TWs. The findings indicate that students perceived key aspects such as the joint acquisition of new knowledge, understanding of profession-specific content, and the development of interprofessional skills as central to IPL. Within the teaching setting, profession-specific content was covered to varying extents, with practice-oriented elements such as case studies and shadowing experiences strengthening the connection to real-world practice. The collaboration among the different professions was at times multiprofessional and at other times truly interprofessional. Facilitating factors such as practice-oriented teaching methods and effective guidance from instructors were contrasted by challenges like unequal participation among professions and a lack of dedicated time for exchange. The perceived learning gains included a better understanding of profession-specific content, leading to an improved understanding of professional roles. There was also a noted increase in interprofessional competencies, such as greater appreciation for other professions and the ability to integrate perspectives from different disciplines. Students voiced specific suggestions for improvement, including allocating more time in the curriculum for interprofessional exchange, achieving a more balanced distribution of content, and receiving more in-depth input from instructors. The results underscore that IPL does not occur automatically but requires intentional opportunities and structural support in order to succeed. This is also supported by Zwaan et al. in their integrative review on barriers and facilitators of IPL [25]. For example, opportunities for communication with other professional groups and practice-related content connected to the future workplace support IPL, whereas time constraints pose a barrier.

Collaboration on shared goals in teaching was addressed by participants in this study, as well as in other mixed-methods studies [9], [13], [14]. In the literature, a distinction is made between multi- and interprofessional collaboration [1]. According to the students, some tasks in the TWs were designed to be completed through IPC. However, these tasks were often completed individually and only merged at the end, without any exchange or direct collaboration taking place. This description aligns more closely with the definition of multiprofessional rather than interprofessional collaboration [1]. In the future, shared time slots for working on tasks, as well as assignments that specifically require inter- rather than monoprofessional collaboration, could help promote IPC. The literature suggests interprofessional group work, in which students independently define a shared interprofessional goal and work together to achieve it [26]. Additionally, interprofessional teaching methods used by instructors – such as co-planning and co-delivering lessons – can actively foster interprofessional exchange [26].

Several of the factors identified by the students in this study as supportive of IPL are consistent with findings from other studies. Practical content, sufficient time for exchange, group work, and adequate input from instructors have also been described in other studies as beneficial for IPL, highlighting the importance of these factors [11], [12], [13], [14], [15], [26]. Challenges mentioned by students in this study – such as imbalances between professions, unequal representation of profession-specific content, lack of professional diversity within groups, and varying levels of prior knowledge – have also been discussed in previous research [11], [12]. For instance, an imbalance in the contributions and content from different professions, as well as an uneven distribution of professions in group work, can lead to some perspectives being underrepresented [12].

A positive aspect emphasized in various studies is the reduction of inhibitions and the increased appreciation for other professions through IPL [10], [16]. Similarly, students in the present study reported a greater understanding and respect for the skills and perspectives of other healthcare professions. These findings reaffirm the relevance of IPL as preparation for effective IPC in professional practice [2].

The insights gained from this study offer points of reference beyond the specific context of the TWs for other health professions and interprofessional teaching formats – provided that comparable structural and didactic conditions are in place. It should be noted that the participating students had already completed vocational training. Initial practical experiences with IPC may have influenced their perceptions and expectations of the teaching format, representing a relevant contextual factor that could limit the transferability of the findings to other learning settings. At the same time, these very practical experiences could enrich the teaching context – for example, by enabling students to participate with more concrete expectations and reference points – making the results particularly relevant for advanced degree programs targeting individuals with prior professional qualifications.

One limitation of this study is that the sample consisted exclusively of female participants, which may restrict the generalizability of the results – although the predominantly female composition of therapeutic professions partially mitigates this limitation. In the second phase, a balanced composition of the focus groups in terms of professional background was sought, in line with the principle of perspective triangulation, to ensure that no profession was over- or underrepresented. To encourage active participation and account for group dynamics, the size of the focus groups was kept small, and one focus group interview was conducted per TW. Technical issues during virtual focus group interviews occasionally required asynchronous communication, which may have distorted some of the responses. Regarding the episodic interviews in phase 1, it should be noted that data collection was conducted by inexperienced student researchers. Additionally, the dual role of the students as both researchers and participants in the TWs may have introduced bias. Despite documented reflection processes, the possibility of unintended influence on data collection and interpretation cannot be entirely ruled out. Supplementary free-text responses from the central course evaluation were also included, providing additional perspectives. However, they did not contain systematic information – such as professional affiliation – limiting the precise description of the sample in this part of the study. Nevertheless, they served as a valuable basis for developing the focus group interview guide used in the second phase. Future studies should include a greater diversity of perspectives and additional interprofessional modules from various higher education institutions, involving further student cohorts and incorporating the perspectives of teaching staff. A theory-generating research approach such as grounded theory methodology [27] could facilitate the development of a theory of IPL in the therapy professions that is applicable across different academic contexts.

## Conclusion

Through their descriptions of the course implementation and the suggestions they expressed, the students indicated that the collaboration in the TWs was predominantly multiprofessional rather than truly interprofessional. A revision of the interprofessional teaching concept, both in terms of content and organization, appears advisable. Allocating sufficient time resources for both instructors and students could be beneficial for IPL as well as IPC in the context of student task work. Moreover, it becomes evident that IPL processes do not arise automatically – they must be intentionally initiated and supported through appropriate didactic and organizational measures.

## Abbreviations

- GDPR: General Data Protection Regulation
- IPL: Interprofessional Learning
- IPZ: Interprofessional Collaboration
- TW: Topical Workshop
- SRQR: Standards for Reporting Qualitative Research
- UzL: Universität zu Lübeck

## Authors’ ORCIDs

- Marie Jacob: [0009-0009-6244-0726]
- Kerstin Lüdtke: [0000-0002-7308-5469]
- Katharina Röse: [0000-0002-9647-7035]

## Acknowledgements

We would like to thank the students of the additive bachelor’s program in occupational therapy/speech and language therapy, who collected the initial data as part of a student research project. We also thank the state of Schleswig-Holstein for its financial support through the open access publication fund program.

## Competing interests

The authors declare that they have no competing interests. 

## Supplementary Material

Focus group guidelines

Supplementary questionnaire

Category manual (final version)

## Figures and Tables

**Table 1:**
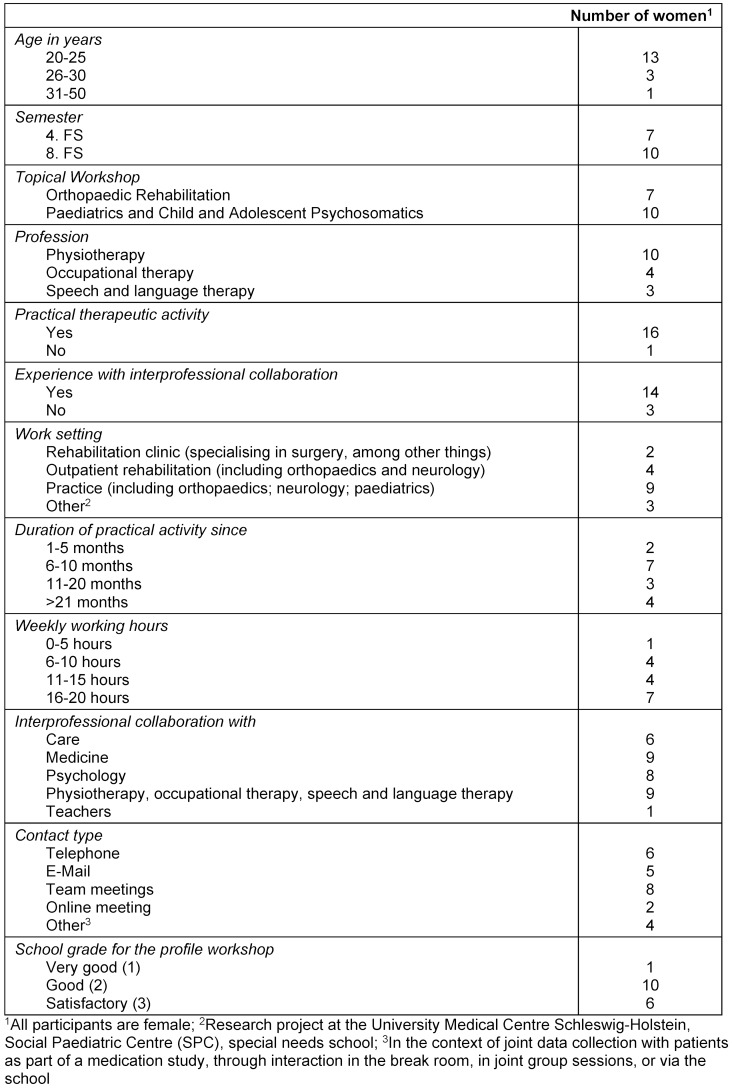
Sample description
