# Supplementary material for: Interprofessional learning in the topical workshops at the Universität zu Lübeck – a qualitative study
Source: GMS J Med Educ. 2025 Nov 17;42(5):Doc64. doi: 10.3205/zma001788 (PMC12661560; doi:10.3205/zma001788)
Supplement: Focus group guidelines [file JME-42-64-s-001.pdf]

## Attachment 1: Focus group guidelines

UNIVERSITÄT ZU LÜBECK
INSTITUT FÜR GESUNDHEITSWISSENSCHAFTEN

**Focus group guidelines for the master's thesis "interprofessional learning in the topical workshops at the University of Lübeck"**

Version 07 July 2023

Introductory Text:

We will begin with a short welcome round. Please introduce yourselves by stating your name and your profession. This will help me later during transcription to assign voices to speakers. Of course, your privacy will be always respected.

Thank you again. The focus group will last approximately 60 to 90 minutes.

You have already received and signed the information sheet and consent form, and you have completed the brief questionnaire. You have also been informed that the session will be recorded and that the data will be processed by me as part of the study.

Before we begin, do you have any further questions?

This project is about the different perspectives of students at the University of Lübeck who took part in an interprofessional Topical Workshop in the winter semester 22/23.

I will now ask you a series of questions about your specific workshop and your experiences. I am particularly interested in your personal reflections and perceptions regarding interprofessional learning. I have prepared a guideline to help structure our discussion, and I may take brief notes so that I can follow up with further questions if needed.

I would like to clarify a few points in advance regarding how the discussion will be conducted:

- Everything shared in this group will be treated confidentially.
- Everyone is encouraged and welcome to contribute.
- Please allow one another to finish speaking.
- There are no right or wrong answers.
- If I notice that a topic is being discussed in great depth or at length, I may gently intervene to move on to the next question to stay within the overall time frame.

Do you have any questions about the procedure before we begin?

Attachment 1 to Jacob M, Lüdtke K, Röse K. *Interprofessional learning in the topical workshops at the Universität zu Lübeck – a qualitative study.* GMS J Med Educ. 2025;42(5):Doc64. DOI: 10.3205/zma001788

| Topic addressed | Interview question | Question | Notes/ Answered? |
|---|---|---|---|
| Introduction to the interview | 1. To begin, please take a moment to recall the Topcial Workshop XY that you attended during the winter semester. How did this module go? | 1.1 What were the key contents of the module?<br><br>1.2 What was the general mood of the module?<br><br>1.3 What did you particularly like?<br><br>1.4 What did you not like? | ☐<br><br>☐<br><br>☐ |
| Subjective understanding of interprofessional learning | 2. A central aspect of the Topical Workshops was their interprofessional nature—you attended and learned together as speech and language therapists, occupational therapists, and physiotherapists. What do you personally understand by interprofessional learning? | 2.1 What elements are essential to interprofessional learning? | ☐ |
| Implementation of interprofessional learning in the Topical Workshop | 3. Now I'd like to take a closer look at the learning experience in your Topical Workshop. How was interprofessional learning implemented in your workshop? What do you remember most? Please feel free to describe example situations.<br><br>4. How were the different professions connected with each other?<br><br>5. In your opinion, what aspects of the Topical Workshop supported or facilitated interprofessional learning? | 3.1 To what extent did you engage with the content of the other professions?<br><br>3.2 To what extent were the assessments used by other professions discussed?<br><br>3.3 To what extent were case studies integrated in the teaching?<br><br>4.1 To what extent were different professional perspectives and methods integrated?<br><br>4.2 In which situations did interprofessional collaboration actually take place?<br><br>5.1 What helped you personally to grow in terms of interprofessional learning? | ☐<br><br>☐<br><br>☐<br><br>☐<br><br>☐<br><br>☐ |

Attachment 1 to Jacob M, Lüdtke K, Röse K. *Interprofessional learning in the topical workshops at the Universität zu Lübeck – a qualitative study.* GMS J Med Educ. 2025;42(5):Doc64. DOI: 10.3205/zma001788

| Topic addressed | Interview question | Question | Notes/ Answered? |
|---|---|---|---|
| | 6. What did you experience as obstacles or challenges to interprofessional learning in the Topical Workshop? | 6.1 What made interprofessional learning more difficult or hindered your personal development? | ☐ |
| Practical relevance | 7. What practical components were included in your Topical Workshop, and how were they organised? Can you give an example that illustrates this for me? | 7.1 How did you experience the work shadowing element of the Topical Workshop?<br><br>7.2 What role did the practical components – such as work shadowing, case study work, or assessment testing – play in terms of interprofessional learning? | ☐<br><br>☐ |
| Subjectively perceived learning gain | 8. What knowledge or skills from the Topical Workshop do you consider relevant for your future work as a therapist?<br><br>9. What aspects of interprofessional learning in the Topical Workshop do you feel prepared you for future collaboration with other healthcare professions? | 8.1 To what extent were you able to apply knowledge from another profession in the context of the workshop?<br><br>9.1 To what extent were you able to integrate knowledge from another profession into your own professional perspective?<br><br>9.2 To what extent did the workshop help you define and differentiate the responsibilities of the various professions? | ☐<br><br>☐<br><br>☐ |
| Wishes | 10. If you had the opportunity to design the Topical Workshop yourself – with plenty of time and resources – what would the teaching look like? | 10.1 What suggestions or wishes do you have for optimising the Topical Workshop in future semesters?<br><br>10.2 What ideas do you have for improving interprofessional teaching, if the unequal number of students per profession remains unchanged? | ☐<br><br>☐ |
| Re-election | 11. Under what circumstances would you choose to take the Topical Workshop again? | 11.1 Why would you vote for the Topical Workshop again / not again? | ☐ |

| Topic addressed | Interview question | Question | Notes/<br>Answered? |
|---|---|---|---|
| Other questions | 12. is there anything else we haven't talked about yet that you would like to add? | | ☐ |
