# Supplementary material for: Interprofessional learning in the topical workshops at the Universität zu Lübeck – a qualitative study
Source: GMS J Med Educ. 2025 Nov 17;42(5):Doc64. doi: 10.3205/zma001788 (PMC12661560; doi:10.3205/zma001788)
Supplement: Supplementary questionnaire [file JME-42-64-s-002.pdf]

Attachment 2: Supplementary questionnaire

UNIVERSITÄT ZU LÜBECK
INSTITUT FÜR GESUNDHEITSWISSENSCHAFTEN

Supplementary Questionnaire for Students of Physiotherapy, Occupational Therapy and Speech and Language Therapy at the University of Lübeck

Thank you for taking part in the study "Interprofessional Learning in the Topical Workshops". To help us gain a better understanding of your academic and professional background, please answer the following questions. Kindly return the completed questionnaire to me by ____________________.

Date: |__|__|Day|__|__|Month|__|__|__|__|Year

ID: ______________________________

Age: ____________________________

Gender: ☐ Female ☐ Male ☐ Diverse

In which semester are you studying? _________________________

Which Topical Workshop did you take part in?

☐ Orthopaedic Rehabilitation

☐ Paediatrics and Child and Adolescent Psychosomatics

Which profession do you belong to?

☐ Physiotherapy

☐ Occupational therapy

☐ Speech and language therapy

Attachment 2 to Jacob M, Lüdtke K, Röse K. *Interprofessional learning in the topical workshops at the Universität zu Lübeck – a qualitative study.* GMS J Med Educ. 2025;42(5):Doc64. DOI: 10.3205/zma001788

Are you currently working in a therapeutic profession?

☐ Yes ☐ No

If yes, in which setting?

☐ Acute clinic
☐ Rehabilitation clinic
☐ Outpatient rehabilitation
☐ Medical practice
☐ Other:____________________________________(please specify)

(Please also specify the speciality):

____

If yes, since when: ___________________________

☐ Full-time ☐ Part-time ☐ Marginal employment

Hours worked per week: ________

Do you already have experience working in an interprofessional team?

☐ Yes ☐ No

If yes, which professional groups do you work with / have you already worked with?

☐ Nursing
☐ Medicine
☐ Psychology
☐ Physiotherapy
☐ Speech therapy
☐ Occupational therapy
☐ Midwives
☐ Others: __________________ (please specify)

Attachment 2 to Jacob M, Lüdtke K, Röse K. *Interprofessional learning in the topical workshops at the Universität zu Lübeck – a qualitative study.* GMS J Med Educ. 2025;42(5):Doc64. DOI: 10.3205/zma001788

How do you liaise with other professional groups?

- ☐ By telephone
- ☐ By e-mail
- ☐ Team meeting
- ☐ Supervision
- ☐ Online meeting
- ☐ Fax
- ☐ No contact
- ☐ Other: __________________ (please specify)

What grade (1 - very good to 6 - unsatisfactory) would you give the Topical Workshop?

1	2	3	4	5	6

The data you provide will be anonymised and treated in strict confidence!

I would like to thank you for your co-operation and will be happy to answer any questions you may have. Please contact me at m.jacob@uni-luebeck.de.

Marie Jacob

Attachment 2 to Jacob M, Lüdtke K, Röse K. *Interprofessional learning in the topical workshops at the Universität zu Lübeck – a qualitative study.* GMS J Med Educ. 2025;42(5):Doc64. DOI: 10.3205/zma001788
