# Supplementary material for: Interprofessional learning in the topical workshops at the Universität zu Lübeck – a qualitative study
Source: GMS J Med Educ. 2025 Nov 17;42(5):Doc64. doi: 10.3205/zma001788 (PMC12661560; doi:10.3205/zma001788)
Supplement: Category manual (final version) [file JME-42-64-s-003.pdf]

Attachment 3: Category manual (final version)

Presentation of the main categories

Main category	Definition of	Anchor example
Main Category 1: Subjective understanding of interprofessional learning	Individual ideas and descriptions of what respondents understand by interprofessional learning.	"Erm. I would start. Um so for me it means that um all disciplines, disciplines, share their knowledge, what you have subject-specific um with each other. Um yes, that the knowledge is simply broader of everything. So whether it's the development in the first years of life under the different aspects of the therapeutic professions um and so theory and practice, so practice, experiences and perspectives um are exchanged precisely." (F5, ll. 191-196)<br><br>"For me personally, interprofessional learning means first and foremost that you learn together, that you exchange ideas and learn something from the other professions. So what does the professional group actually do and what techniques are there perhaps, so that you have a rough insight into what the professional group does, what makes it special and that you can better assess this later in interdisciplinary cooperation or interprofessional cooperation, um, that everyone knows what the task of the respective profession ultimately is." (F2, ll. 118-125)<br><br>"Of course, taking courses together in any case. (Laughter) I think that's a basic requirement. And that a standardised language is used so that you can understand the other profession when you talk to each other. And that people are simply taught how to communicate with each other, including which areas of work are covered by which profession. In other words, where the boundaries of the professions lie so that you can intercept each other, so to speak. That you simply get to know each other's areas of work." (I1, ll. 105-113)

Main category	Definition of	Anchor example
Main category 2: Implementation of interprofessional learning in teaching	Descriptions of how interprofessional learning was implemented within the courses at the university and which elements supported interprofessional learning and which made it more difficult.	"Well, first of all, there were just as many parts that were led by the occupational therapists, i.e. the event was now carried out, guided, so to speak, as by the physiotherapists. And um then it was just about the fact that the structure of such a lecture was the same in each case, but um different focal points or both focal points, i.e. physiotherapy and occupational therapy, were highlighted in the um diagnostics and also in the treatment. In other words, tests and assessments from occupational therapy and physiotherapy were presented and then a physiotherapy or occupational therapy treatment plan was presented. And we tried to make sure that it was equal and that/ Yes, that both professional groups had an equal say. And when it turned into a free discussion, both professional groups were always asked to contribute in some way." (F1, ll. 169-180)<br><br>"I would start from the beginning. Well, I'd say that the fact that the groups were so spread out meant that there was a lot of dialogue. In my opinion, it was always a bit difficult because we were so different in terms of numbers that there were more physios in one group than speech therapists and occupational therapists. Erm. That perhaps the focus was sometimes not so much on the exchange. So the interpersonal/interprofessional exchange was more among themselves. And I had the feeling that the tasks were often subdivided, that the professions split up to work on them and didn't think about them together." (F8, ll. 285-293)<br><br>"In other words, we talked about speech therapy, for example, how the development is naturally in the different years of life. And then in the next lesson, for example, we looked at occupational therapy tests, such as this one (MABC test?) or which ones, so we really looked at a lot of different tests. And I think we even looked at studies, at reliability and exactly how reliable they are." (I5, ll.104-112)

Main category	Definition of	Anchor example
Establishing practical references	Descriptions of how the practical relevance of what has been learnt within the module has been established.	“During my work shadowing, I was also able to take a look at physiotherapy and speech therapy, but I was lucky that it was actually an interdisciplinary early intervention programme. That means I also got to see the dialogue between all of them, so to speak. It was actually more or less like the case discussion that we had in real life, so to speak.” (F8, ll. 806-810)<br><br>“Okay, then I would definitely say that we, the, yes, the majority of the assessments were always carried out. So it was always the case that those who presented the tests always presented them and then we had the opportunity to try them out and that was also a practical part for me. But also the case studies that we then had, which were just a bit more practical, where you could perhaps visualise it a bit better. I would also count that um, yes, under the practical part, because it made it easier to transfer.” (F2, ll. 535-543)<br><br>“So a very large part of the practical part was of course the work placement that we had to do. This allowed us to really experience what we were told and then these case studies, which were also a bit more practical, so we sat together and discussed them and didn't just listen to them, so to speak, and with the assessments it was also a practical part, so to speak, that we actually had the assessments in our hands (...) we didn't actually do it properly, but we did get to grips with it a bit and then this video, which was particularly good when we watched it and then filled in the assessment afterwards and then discussed who chose what, which was also really good to really internalise that, how do I rate it now and how not, exactly.” (F3, ll. 160-170)

Main category	Definition of	Anchor example
Main category 3:<br>Subjectively perceived learning gains	Descriptions of the areas in which students perceive a successful or unsuccessful learning gain as a result of the module.	“Well, I would say the approach is definitely the right one. I had already touched on this a bit before, that you should perhaps take an all-encompassing look at the patient's environment, ask about it and somehow have a better understanding of what else might be relevant for the patient. What you might also need to ask about, in any case. And yes, that you can also somehow define more clearly what occupational therapy actually does with the patients and what things I can then ask them about and also, um, what things I can then advise the patient to ask the other profession about again, that this falls within their area of responsibility and that you can then define this better from each other, I would definitely say that.” (F1, ll. 703-713)<br><br>“To be introduced to cooperation at an early stage so that the fear of contact and overcoming the barriers to exchange are not so great.” (F10, ll. 987-989)<br><br>“I thought that was a bit of a shame because somehow I didn't really learn how to use this assessment. For example, I now know the name and what it's supposed to do, but it's just this thing again of how do I really put it into practice, because even in my training we got to know a lot of assessments, but somehow never really applied them and when you apply them in practice for the first time, it’s kind of stupid, because then mistakes happen and you're nervous because it's not yet routine.” (I4, ll. 80-87)
Main category 4:<br>Wishes	Based on the descriptions of the module and the subjective learning gain, wishes and suggestions for improvement for the further development of the module are described.	“I was also just thinking about another point that hasn’t been mentioned yet, which I would also find very interesting, would be what research looks like in the respective professions. So, um, in general, what kind of research already exists and how it is implemented in the professions, because I think there are a lot of differences here.” (F1, ll. 897-901)<br><br>“I think I would choose lecturers in equal numbers. So not most of them from Ergo, but somehow, um that it’s relatively balanced, that all three professions, since female research assistants or professors are somehow involved in teaching, I think that would also bring a bit of um, yes, more different perspectives um in teaching, exactly that. I think that would be the first thing I would do.” (F13, ll. 1034-1039)<br><br>“That’s a good question. (Um) Well, for one thing, I think it would be totally desirable or cool if other professions like nursing or medicine were involved.” (I1, ll. 471-473)

Presentation of the subcategories

Main category 1: Subjective understanding of interprofessional learning

Subcategory	Definition of	Examples from the data material
Learning new things together	This category contains statements that the joint learning of content that is new for all participating professions is relevant for interprofessional learning.	- Participants attend joint events in which content is learnt that is new to all professions.<br>- Joint learning takes place from the very beginning.<br>- Practice is carried out jointly.
Understanding profession-specific content	This category contains statements that an understanding of the content of the other professions is a central aspect of interprofessional learning.	- Profession-specific content is learnt from one another.<br>- In the context of improved role clarification, it is important to recognise the boundaries of one's own professional group and to understand the tasks of other professional groups.<br>- Similarities and differences between the professional groups are recognised and can be explained to patients/clients.
Interprofessional skills	This category includes statements on which skills are addressed as part of the interprofessional learning process.	- The exchange of opinions between different therapeutic professions is important in the context of interprofessional learning in order to obtain different perspectives and approaches to solutions and to broaden one's own horizons.<br>- There is consistency of language between the professions.<br>- Work is carried out towards a common goal.

Main category 2: Implementation of interprofessional learning in teaching

Subcategory	Definition of	Examples from the data material
Involvement of the professions in teaching to varying degrees	This category contains statements on the extent to which the professions were included in the courses in equal proportions.	- Information from the various professions was imparted in the courses.<br>- There were lecturers from every profession, with occupational therapy having the largest share in the topical workshop for paediatrics and child and adolescent psychosomatics.<br>- In the topical workshop for orthopaedic rehabilitation, an attempt was made to involve both professional groups equally and the lecturers in the courses increasingly tried to obtain contributions from the occupational therapists in order to shed light on both perspectives in diagnostics and treatment. Nevertheless, the physiotherapy components were superior to the occupational therapy components.
Familiarisation with profession-specific content	This category includes statements on which content of the other professions was thematised in which didactic manner.	- Theoretical principles, assessments, treatment methods and concepts as well as procedures and aids were discussed.<br>- The profession-specific content was taught through simulations such as treatment simulations with video accompaniment or simulated case conferences, through free exploration of games as a therapy medium, through group lectures, through the use of case studies, through joint practice and through contributions from the lecturers within the courses.
Linking the professions	This category contains statements on the extent to which the content of different professions has been linked.	- The professions were linked, for example, by the lecturers, who organised the teaching in a team-teaching format, through simulated case conferences, through presentations by the students and within the work shadowing sessions.<br>- In some cases, the lack of dialogue made it difficult to link the professions.
Collaboration	This category includes statements on the extent to which interprofessional collaboration has taken place.	- Cooperation between the professions took place, for example, in the context of work shadowing and through free exploration of the games within the course.

		- Some of the case studies were worked on together, but in some cases the tasks were merely distributed and collated at the end, meaning that there was no direct collaboration.
Establishing practical references	This category contains statements that practical references were made both inside and outside the courses as part of the module.	- Within the courses, the use of case studies, the testing of assessments, the use of simulations and the presentation of aids served to convey content in a more practical way.<br>- The theoretical content learnt was transferred into practice during work shadowing in another therapeutic profession.<br>- The experiences from the work shadowing sessions at the topical workshop for orthopaedic rehabilitation served as the basis for case studies, which were integrated into a student presentation to facilitate practical relevance.<br>- In some cases, students shadowed in their own therapeutic profession and not in another one, as it was difficult for them to organise places to do so.
Facilitating factors	This category includes statements about the resources available for interprofessional learning in this module.	- Practical content such as carrying out assessments, an excursion, the presentation of aids, movement analysis, treatment simulation in the form of skills training, the use of video materials, work shadowing, simulated case conferences, exploring games and working on case studies was supportive.<br>- Free tasks that allow sufficient room for interprofessional exchange were supportive.<br>- Group work in interprofessional teams was supportive.<br>- The team teaching and the good guidance as well as the content-related contributions of the lecturers were supportive.<br>- A good module structure including a common thread running through the module and a variety of teaching methods was supportive.<br>- Lectures such as certain lecture series and guest lectures were supportive.<br>- An open attitude and curiosity of the students were supportive.

Subcategory	Definition of	Examples from the data material
Challenging factors	This category includes statements on the challenges that existed for interprofessional learning in this module.	- Online teaching or working on content alone at home was challenging.<br>- The lack of exchange opportunities within the framework of the courses was challenging.<br>- Too few contributions in terms of content from the lecturers and insufficient agreements between the lecturers and with the students were challenging.<br>- The unequal distribution of students from different professions (professional homogeneity), the unequal content-related representation of the professions, and the lack of professional diversity in group work were challenging.<br>- The unequal level of knowledge between students from different professions was challenging.<br>- The lack of support from lecturers in organising work shadowing placements was challenging.<br>- The tasks that addressed intraprofessional exchange and not interprofessional exchange and the different information content for the various professions within case studies were challenging.

<u>Main category 3</u>: Subjectively perceived learning gains

Subcategory	Definition of	Examples from the data material
Successful learning gain		
Understanding of profession-specific content	This category contains statements on the basic professional knowledge acquired by the students.	- The repetition of knowledge is perceived as enriching, especially in the cohort from the 2022/23 semester, as the repetition facilitates career entry and offers the opportunity to ask questions and practice practically.<br>- The students have recognised that their work is often similar and at the same time have learned to recognise the boundaries and differences between the various professions and have thus gained an improved understanding of the roles of the professions.<br>- Assessments and treatment methods as well as the theoretical foundations of the various professions were internalised.
Interprofessional skills	This category contains statements on the interprofessional skills developed through the Topical Workshops.	- Interprofessional learning within the topical workshop helped to break down inhibitions about entering into dialogue with other professions.<br>- The potential for complementarity within the framework of interprofessional collaboration was recognised.<br>- Appreciation for the other professions with their respective areas of responsibility and perspectives was increased.<br>- The perspectives of other professions were integrated into our own thinking.
Unsuccessful learning gain		
Understanding of profession-specific content	This category includes statements on the extent to which learning gains were inadequate and which profession-specific basic knowledge the students lack after completing the Topical Workshop.	- The repetition of knowledge is perceived as disruptive and unhelpful, especially in the cohort from the 2021/22 semester; the students perceive the learning gain between the professions as unequal.<br>- In some cases, assessments were only discussed and not carried out practically, which meant that students did not feel well prepared for the practical implementation of assessments.

Unsuccessful learning gain		
Interprofessional skills	This category contains statements on which interprofessional skills were not developed through the topical workshops.	- Content from other therapeutic professions is not used or applied in their own therapeutic setting.

Attachment 3 to Jacob M, Lüdtke K, Röse K. *Interprofessional learning in the topical workshops at the Universität zu Lübeck – a qualitative study.* GMS J Med Educ. 2025;42(5):Doc64. DOI: 10.3205/zma001788

<u>Main category 4</u>: Wishes

Subcategory	Definition of	Examples from the data material
In terms of content		
More practical relevance	This category includes expressed wishes for more practical relevance within the module.	- The excursion into orthopaedic technology should be intensified.<br>- The processing of case studies should be intensified.<br>- The movement analysis in the topical workshop for paediatrics and child and adolescent psychosomatics should be intensified.<br>- The quality and quantity of the case conferences should be intensified.
Increased role clarification	This category contains statements that the clarification of roles between the professions should be focussed on even more.	- An event in which the various professions are presented and similarities and differences are explored was suggested.
Freer task definition & group mixing	This category includes expressed wishes for freer and group-mixing tasks.	- The tasks should have less strict time and content requirements so that there is more time and space for interprofessional dialogue.<br>- The tasks should be designed to be more interprofessional, so that there can be a mix of professions and thus more interprofessional exchange within the groups.
More space for interprofessional exchange	This category contains statements that an increased interprofessional exchange is desired.	- The exchange with lecturers should be intensified in order to benefit from their experience.<br>- Discussions of challenges in everyday practice should take place through an interprofessional exchange.<br>- More interprofessional partner or group work should be carried out.<br>- An increased interprofessional exchange should take place within the framework of fictitious case discussions.<br>- More time should be planned for exchange between students.

Subcategory	Definition of	Examples from the data material
In terms of content		
Optimised presentation of case studies	This category includes statements that the case studies used should be optimised in some areas.	- The case studies should be illustrated with video material.<br>- The case studies should have the same information content for all professions.<br>- The case studies should be as realistic as possible.
Reduction/modification of certain content	This category contains requests to reduce or change certain contents of the module.	- The games day should be shortened.<br>- The skills training should include fewer passes.<br>- The duration of the work shadowing days should be shortened.
Familiarisation with profession-specific research	This category contains statements about the desire to familiarise oneself with profession-specific research.	- Profession-specific research, including the current state of research and the study designs used, should be thematised.<br>- Differences in research between the professions should be addressed.
More input from lecturers	This category contains statements that there is a desire for more input from the lecturer.	- The contribution of real-life examples of successful interprofessional collaboration by lecturers was considered helpful.<br>- Increased contributions by lecturers on the links between the professions and possible areas of interprofessional encounters were desired.
Organisational		
Better exchange between lecturers	This category contains expressed wishes for a better exchange between lecturers.	- Agreements between lecturers should be improved so that responsibilities are clear and discrepancies in the execution of tasks can be avoided.
Better organisation & reflection of the job shadowing	This category includes expressed wishes for better organisation and reflection of the work shadowing sessions.	- Lecturers should support students in finding work shadowing places so that all students have the opportunity to work shadow in another profession.<br>- The work shadowing should be accompanied and subsequently reflected on within the course.

Organisational		
Recording of students' prior knowledge & wishes	This category contains statements that students' prior knowledge, goals and wishes should be recorded at the beginning of the module.	- The students' previous knowledge should be recorded so that content is not repeated too often, so that there can be a balanced learning progression between the different professions and so that no one is questioned about experiences that they have not yet had.<br>- The goals and wishes of the students should be recorded so that these can be addressed within the courses.
Balance of professions	This category contains statements that the number of representatives of the various professions should be balanced.	- The number of students from different professions should be balanced.<br>- The number of lecturers from different professions should also be balanced.
Free choice of topical workshop	This category includes statements to the effect that the choice of Topical Workshop must be voluntary.	- A free choice of topical workshops according to personal interests should be maintained.
Balanced content for all professions	This category includes statements that the content of the different professions should be balanced.	- There is a need for more speech therapy and physiotherapy content in the topical workshop for paediatrics and child and adolescent psychosomatics.<br>- There is a need for more occupational therapy content in the topical workshop for orthopaedic rehabilitation.
Inclusion of other people	This category includes expressed wishes to include further groups of people in the module.	- Students from other professions, such as nursing or medicine, should be included.<br>- Other lecturers, such as external lecturers who work in practical interprofessional settings, should be involved.
